# Downregulation of Mir-31, Mir-155, and Mir-564 in Chronic Myeloid Leukemia Cells

**DOI:** 10.1371/journal.pone.0035501

**Published:** 2012-04-12

**Authors:** Oshrat Hershkovitz Rokah, Galit Granot, Adelina Ovcharenko, Shira Modai, Metsada Pasmanik-Chor, Amos Toren, Noam Shomron, Ofer Shpilberg

**Affiliations:** 1 Felsenstein Medical Research Center, Beilinson Hospital, Sackler School of Medicine, Tel Aviv University, Tel Aviv, Israel; 2 Department of Cell and Developmental Biology, Sackler School of Medicine, Tel Aviv University, Tel Aviv, Israel; 3 Bioinformatics Unit, Faculty of Life Sciences, Tel Aviv University, Tel Aviv, Israel; 4 Department of Pediatric Hematology-Oncology, Safra Children's Hospital, Tel-Hashomer, Israel; 5 Institute of Hematology, Beilinson Hospital, Sackler School of Medicine, Tel Aviv University, Tel Aviv, Israel; National Institutes of Health, United States of America

## Abstract

**Background/Aims:**

MicroRNAs (miRNAs) are short non-coding regulatory RNAs that control gene expression and play an important role in cancer development and progression. However, little is known about the role of miRNAs in chronic myeloid leukemia (CML). Our objective is to decipher a miRNA expression signature associated with CML and to determine potential target genes and signaling pathways affected by these signature miRNAs.

**Results:**

Using miRNA microarrays and miRNA real-time PCR we characterized the miRNAs expression profile of CML cell lines and patients in reference to non-CML cell lines and healthy blood. Of all miRNAs tested, miR-31, miR-155, and miR-564 were down-regulated in CML cells. Down-regulation of these miRNAs was dependent on BCR-ABL activity. We next analyzed predicted targets and affected pathways of the deregulated miRNAs. As expected, in K562 cells, the expression of several of these targets was inverted to that of the miRNA putatively regulating them. Reassuringly, the analysis identified CML as the main disease associated with these miRNAs. MAPK, ErbB, mammalian target of rapamycin (mTOR) and vascular endothelial growth factor (VEGF) were the main molecular pathways related with these expression patterns. Utilizing Venn diagrams we found appreciable overlap between the CML-related miRNAs and the signaling pathways-related miRNAs.

**Conclusions:**

The miRNAs identified in this study might offer a pivotal role in CML. Nevertheless, while these data point to a central disease, the precise molecular pathway/s targeted by these miRNAs is variable implying a high level of complexity of miRNA target selection and regulation. These deregulated miRNAs highlight new candidate gene targets allowing for a better understanding of the molecular mechanism underlying the development of CML, and propose possible new avenues for therapeutic treatment.

## Introduction

Chronic myeloid leukemia (CML) is one of the most extensively studied and, probably, best understood neoplasms. The cytogenetic hallmark of CML is the Philadelphia chromosome (Ph), created by a reciprocal translocation between chromosomes 9 and 22 (t[9]; [22] [q34;q11]). This translocation results in the formation of a hybrid bcr-abl oncogene on chromosome 22, which codes for a deregulated tyrosine kinase. BCR-ABL activates multiple signal transduction pathways, including mitogen-activated protein kinase (MAPK), phosphatidylinositol 3 kinase, STAT5/Janus kinase, and Myc. BCR-ABL activity leads to uncontrolled cell proliferation and reduced apoptosis, resulting in the malignant expansion of pluripotent stem cells in bone marrow [1]. Since CML is caused by this distinct genetic lesion it was possible to design an effective targeted molecular therapy which selectively inhibits the aberrant BCR-ABL tyrosine kinase. Imatinib mesylate (STI-571 or Gleevec), is the first BCR-ABL tyrosine kinase inhibitor (TKI) to be used for the treatment of CML [2]. Imatinib is a small-molecule drug that competitively binds the ATP-binding site of BCR-ABL thus preventing a conformational switch to the active form of the oncoprotein. This inhibits BCR-ABL autophosphorylation, interferes with its activation and blocks its downstream signal transduction.

MicroRNAs (miRNAs) are a family of 19–24 nucleotide non-coding RNAs, which affect the regulation of gene expression in eukaryotic cells by binding to a 3′-untranslated region (3′ UTR) within target messenger RNAs (mRNAs). MiRNAs are transcribed by RNA polymerase II as long primary transcripts (pri-miRNAs) and undergo sequential processing to produce mature miRNAs [3], [4]. MiRNAs play important roles in many cellular processes such as development [5], stem cell division [6], [7], apoptosis [8], [9] and cancer [10], [11]. MiRNAs regulate gene expression by either inhibiting translation or promoting degradation of specific mRNA transcripts. An estimated 3% of human genes code for miRNAs, yet these miRNAs may regulate around 30% of the protein-coding genes [12]. This suggests not only their importance in various regulatory pathways, but also their potential for manipulation. MiRNAs themselves have been shown to act both as tumor suppressors and as oncogenes, which can promote tumor growth. In addition, aberrant miRNA levels, specifically an overall downregulation, is observed in many cancers, as compared to their normal tissue counterparts [13], [14]. Recently, a growing body of evidence has implicated specific miRNAs in the pathogenesis of a variety of solid tumors (ovarian, breast and colorectal cancers among others) and hematological malignancies (chronic lymphocytic leukemia (CLL), B-cell lymphomas, acute promyelocytic leukemias, acute lymphocytic leukemia (ALL) and CML) [15], [16]. Most publications on miRNA expression in CML explore the expression of specific miRNAs [17], [18], [19], [20], [21]. Agirre et al [17] revealed decreased expression of miR-10a in CML-derived CD34^+^ bone marrow cells compared with healthy controls. Venturini et al [18] found increased expression of miR-17-5p in CML-derived CD34^+^ peripheral blood cells though this was disputed by Hussein et al [21] who found no significant difference in the expression of this miRNA in CML-derived bone marrow cells compared to healthy controls. In this same paper, CML-derived cells had a tendency for higher expression levels of miR-155 and miR-223 in a subfraction of cases which was, however, not significant. Overall, the data regarding CML specific miRNA expression is inadequate and sometimes inconclusive and the role of miRNAs in CML is still poorly understood. To better elucidate the significance of differential miRNA expression in CML, it is crucial to examine miRNAs expression profiles through microarray analysis in normal and CML tissues or cell lines.

Here, we profiled miRNAs expressed in the CML cell line K562, as compared to its healthy counterpart, normal blood. With the aid of unsupervised hierarchical clustering we found that healthy blood samples were clustered separately from K562 cells. Following miRNA real-time PCR validation, we have determined that miR-31, miR-155, and miR-564 are downregulated in CML cell lines and patients in comparison to non-CML cell lines and healthy blood. Downregulation of these miRNAs is presumably mediated by BCR-ABL tyrosine kinase activity. Bioinformatics analysis revealed predicted targets and affected pathways of the deregulated miRNAs. As expected, in K562 cells, the expression of several of these targets was inverted to that of the miRNA putatively regulating them. Reassuringly, the analysis identified CML as possibly associated with major deregulation of these miRNAs and MAPK, ErbB, mammalian target of rapamycin (mTOR) and and vascular endothelial growth factor (VEGF) as the main molecular signaling pathways regulated by these miRNAs. Overall, our data indicate that these deregulated miRNAs and their putative targets may play a pivotal role in the biology of CML. Consequently, these miRNAs may serve as potential diagnostic and prognostic biomarkers and with further investigation may be used as possible therapeutics for patients suffering from CML.

## Material and Methods

### Cell lines

The K562 (obtained from the German Resource Center for Biological Material [DSMZ], DSMZ no. ACC 10), Meg-01 (obtained from the American Type Culture Collection [ATCC], no. CRL-2021) and HL60 cell lines (kindly provided by Prof. Shay Izraeli, Sheba Medical Center, Israel) were cultured in RPMI-1640 medium supplemented with 10% heat-inactivated fetal bovine serum (FBS), 2 mM glutamine, 1% penicillin and streptomycin and cultured at 37°C in a humidified incubator with 5% CO2.

The continuous K562 cell line was originally established from the pleural effusion of a 53-year-old female with BCR-ABL positive CML in terminal blast crises. The cell population has been characterized as highly undifferentiated and of the granulocytic series.

The Meg-01 cell line was originally derived from bone marrow cells taken from a patient in megakaryoblastic crisis of BCR-ABL positive CML.

The HL-60 cell line was originally derived from peripheral blood leukocytes of a 36-year-old female with acute promyelocytic leukemia.

### Leukocyte isolation

Peripheral blood samples were drawn, with informed consent, from newly diagnosed CML patients before and on days 5 and 30 of treatment with imatinib at a dose of 100 mg/day on days 1–7, 200 mg/day on days 8–14, 300 mg/day on days 15–21, and 400 mg/day from day 22 and thereafter and from healthy volunteers. The samples were treated with red-blood-cell lysis buffer and the remaining leukocytes were used for further experiments. Only blood samples containing 67–70% granulocytes (according to routine blood counts) were used. Blood samples, meeting these criteria, were mixed with erythrocyte lysis buffer (Qiagen, Germany) and centrifuged at 400 g for 10 min at 4°C. The leukocyte pellet was washed and centrifuged again. The remaining leukocytes were snap-frozen and used for further experiments.

### Ethics statement

All healthy volunteers were members of the Experimental Hematology Lab, Felsenstein Medical Research Center, at the Rabin Medical Center. Before the experiment, participants were provided with verbal information outlining the general purpose of the study and they were informed that they could withdraw at any time without penalty. They were also verbally informed that the data obtained from their samples would be confidential and used only for the purpose of this research. Verbal consent was obtained for this study because the risks were minimal and all participants were associates of the Lab, and were therefore familiar with the basic methods employed in the relevant experiments. The head of the ethics committee; Institutional Review Board (Helsinki committee), Rabin Medical Center, Beilinson Hospital, Petah Tikva, Israel granted us with the following approval in this matter: "Blood sampling from healthy volunteers who are also research workers do not require IRB approval in our country. Therefore I grant you exemption from IRB approval in this matter". Verbal consent was documented according to IRB ethics committee procedures. All methods employed in this study were in accordance with the Declaration of Helsinki.

Studies executed on patient blood samples were performed with consent. The protocols used were approved by the ethics committee; Institutional Review Board (Helsinki committee), Rabin Medical Center, Beilinson Hospital, Petah Tikva, Israel.

### MiRNA microarray analysis

Microarray analysis was performed on the K562 cell line and on a pool of leukocytes derived from blood of 3 healthy volunteers. MiRNAs were extracted from each sample using the miRNeasy Mini Kit (Qiagen, Germany) according to manufacturer's protocol. RNA quantity and quality was assessed using the Nanodrop and Agilent 2100 bioanalyzer systems, respectively. The amount of RNA taken from each sample was equalized, based on these assessments. The miRNA microarray experiments were performed at the Biological Services Unit at the Weizmann Institute of Science using the Agilent Human miRNA Microarray Kit version 1.0 (Agilent Technology, USA). For each miRNA, multiple probes were spotted on the array and the average intensity of these probes was calculated to represent the expression value of the miRNA. In addition, multiple spots were included as negative controls. For each tissue sample, 100ng total RNA was hybridized with the miRNA array and further processed in accordance with the manufacturer's instructions. Two biological replicates were done for each treatment. The arrays were scanned using an Agilent Technology G2565BA scanner and the scanned images were processed using the Feature Extraction software package version 9.5 (Agilent Technology, USA). Coefficient of variation (CV) within groups of replicate probes was used as a quality control measure to reflect the intra-array reproducibility. Complete raw and normalized microarray data and their MIAME compliant metadata have been deposited at GEO (www.ncbi.nlm.nih.gov/geo) under the accession number GSE28825.

### Bioinformatics analysis

#### Partek® genomics suite software

MiRNA microarray results were analyzed using Partek® Genomics Suite software V6.5, Copyright© 2009 (Partek Inc., St. Louis, MO, USA; www.partek.com). Data were normalized and summarized with the robust multi-average method (RMA) [22], using quantile normalization approach [23], followed by Analysis of Variance (ANOVA). Differentially expressed miRNAs were obtained with p<0.05 and fold-change cutoff of 4.

#### MiRNA Target Analysis

Each relevant CML-associated or treatment-associated miRNA was tested using TargetScan. TargetScan uses a computational algorithm to predict biological targets of miRNAs by searching for the presence of conserved 8 mer and 7 mer sites that match the seed region of each miRNA (http://www.targetscan.org) [24]. All the predicted targets from this analysis were then used as input for Ingenuity Pathway Analysis.

#### Ingenuity Pathway Analysis

Ingenuity Pathway Analysis (IPA) identifies lists of genes that satisfy particular biological criteria. IPA scores these genes against pathways in the Ingenuity Knowledge Base which houses a large database of biological and chemical relationships extracted from scientific literature. IPA integrates data from a variety of experimental platforms making it possible to identify signaling and metabolic pathways, molecular networks, and biological/disease processes that are most significantly perturbed in our dataset of interest. For each analysis, IPA ranks each function or pathway using the right-tailed Fisher Exact Test, which measures the likelihood that the lists of genes are associated with a certain pathway/function (www.ingenuity.com).

#### KEGG Pathway Database

The predicted targets from our analysis were also used as input for the KEGG pathway database [25]. KEGG is used as a reference knowledge base for biological interpretation of large-scale datasets generated by high-throughput experimental technologies. KEGG is a collection of manually drawn pathway maps representing our knowledge on the molecular interaction and reaction networks for metabolism, genetic information processing, environmental information processing, cellular processes and human diseases. . KEGG uses in-house software called KegSketch (http://www.genome.jp/kegg/pathway.html).

Utilizing both IPA and KEGG technologies provides clues for the function of our miRNA of interest.

#### Venn diagrams

Venn diagrams were used to show all hypothetically possible logical relations between the top scoring pathways identified by Partek Genomics Suite software V6.5, Copyright© 2009.

### MiRNA-specific real time PCR **(**miR–qRT-PCR**)**

MiRNA real-time RT-PCR was performed using the TaqMan® Small RNA primer and probe sets (Applied Biosystems, USA). RNA was isolated from all cell lines and from leukocytes purified from blood of CML patients and of healthy volunteers. RNA was reverse-transcribed with miRNA specific stem-looped RT primers (Applied Biosystems, USA) and incubated for 30 min at 16°C, 30 min at 42°C and 5 min at 85°C. Real-time PCR was performed in duplicates using the following conditions: 95°C for 10 min, followed by 40 cycles of 95°C for 15 s, and 60°C for 1 min. Each value of miRNA expression is represented relative to the expression of U6-snRNA, which was used as an internal control. The fold change was calculated using 2^−ΔΔCt^ method.

### Real-time PCR

RNA was isolated using RNeasy Plus Mini Kit (Qaigen, Germany) and reverse transcribed using the high capacity cDNA RT kit (Ambion, USA). Real-time PCR was then performed using the SDS 7000 machine (Applied Biosystems, USA) in a 20 µl reaction containing 10 ng RNA, 10 µl TaqMan master mix (Ambion, USA), 1 µl of target gene or GUSB RNA control primers and a FAM dye-labeled TaqMan probe (Ambion, USA). Amplification conditions were: 50°C for 2 min, followed by 95°C for 10 min, then 40 cycles of 95°C for 15 sec and 60°C for 1 min. The ΔΔC_t_ method was used to calculate relative expression levels.

### Statistics

Experiments were done in triplicates. Differences were statistically evaluated by Student's t-test. Unless otherwise stated, p≤0.05 was considered to be statistically significant.

## Results

### Differentially expressed miRNAs in K562 cells

**Figure 1:**
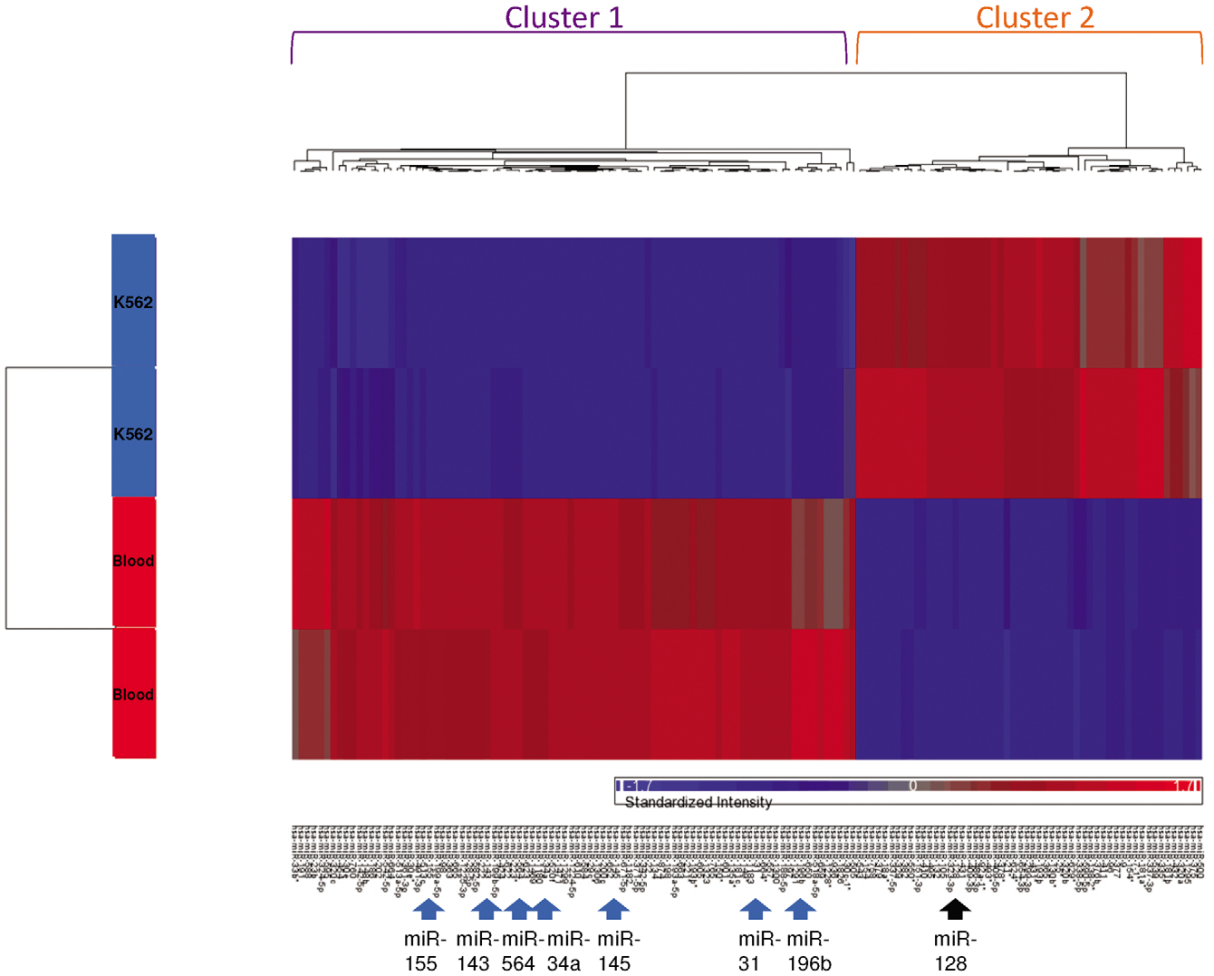
Unsupervised clustering analysis of miRNA expression in K562 cells as compared to a pooled sample of healthy blood. Heat maps illustrating unsupervised clustering of miRNAs that were differentially expressed between blood and K562 samples. The red and blue colors indicate relatively high and low fold-change of expression, respectively. Missing values are indicated in gray. The 8 miRNAs we focused on are indicated with arrows at the bottom of the cluster. Blue arrows represent miRNAs whose expression was downregulated in K562 vs blood, the black arrow represent miRNA whose expression was upregulated in K562 vs blood. Two main miRNAs clusters are shown on top (cluster 1; purple and cluster 2, orange), correlated with the expression patterns described above.

MiRNA expression has been shown to dramatically change in CLL, ALL, lymphoma and in other hematological malignancies [14], [15], [16]. With the objective of deciphering a potential miRNA expression signature associated with CML, we analyzed the miRNAs expression profile of the CML cell line, K562 in reference to a pool of 3 healthy blood samples using a miRNA-based microarray chip assay. Of the 470 mature human miRNAs analyzed, 142 miRNAs showed reasonable expression levels in these cells (see Materials and Methods). We used unsupervised hierarchical clustering to differentiate the CML cell line from the normal tissues (Figure 1). It was encouraging to notice that the healthy blood tissues were clustered separately from the K562 cell line indicating a distinct miRNA expression pattern between these groups. Of the 142 miRNAs remaining in the final analyses; 75 were found to be downregulated in K562 cells as compared to healthy blood samples (Table 1), whereas only 35 were upregulated (Table 2), both with a p-value≤0.009. These data are consistent with previous reports stating that overall downregulation of miRNA expression is characteristic of many cancers, as compared to their normal tissue counterparts [13], [14].

**Table 1:** Summary of miRNAs downregulated in K562 cells as compared to healthy blood samples.

miRNA ID	Fold change	p-value	Raw log2 values (mean K562 vs blood)	miRNA ID	Fold change	p-value	Raw log2 values (mean K562 vs blood)
hsa-miR-584	4E+07	9E−11	0.1 vs 25.16	hsa-miR-513c	53223	0.0006	9.21 vs 24.92
hsa-miR-1299	335610	3E−09	0.1 vs 18.45	hsa-miR-148b	2E+09	0.0002	36.62 vs 67.54
hsa-miR-1471	8E+09	7E−09	0.1 vs 32.92	hsa-miR-491-3p	4	0.0003	0.1 vs 2.24
hcmv-miR-US4	4E+09	8E−09	0.1 vs 32.02	hsa-miR-424	1E+18	0.0008	67.89 vs 129.69
hsa-miR-200c	28	10E−09	0.1 vs 4.89	hsa-miR-371-5p	6E+11	0.0002	0.1 vs 39.19
hsa-miR-520f	23894	1E−08	0.1 vs 14.64	hsa-miR-193b*	285	0.0004	0.1 vs 8.25
hsa-miR-34a	21899	2E−07	0.1 vs 14.51	hsa-miR-150*	2E+16	0.0004	1.38 vs 55.42
hsa-miR-1180	2E+06	2E−07	0.1 vs 20.61	hsa-miR-221*	10	0.0005	0.1 vs 3.39
hsa-miR-623	3E+11	8E−07	0.1 vs 38.30	hsa-miR-671-5p	2E+28	0.0004	1.93 vs 95.69
hsa-miR-1469	24	1E−06	0.1 vs 4.70	hsa-miR-188-5p	3E+22	0.0003	24.39 vs 99.37
hsa-miR-1182	1E+12	4E−06	0.1 vs 40.04	hsa-miR-202	8E+11	0.0010	6.17 vs 45.73
hsa-miR-28-5p	6E+22	8E−06	0.1 vs 75.63	hsa-miR-1323	1966	0.0009	0.1 vs 11.04
hsa-miR-342-5p	1E+20	8E−06	0.1 vs 66.89	hsa-miR-605	41	0.0009	0.1 vs 5.45
hsa-miR-652	9E+25	8E−06	37.45 vs 123.67	hsa-miR-765	8E+37	0.0009	0.1 vs 126.0
hsa-miR-665	417	1E−05	0.1 vs 8.80	hsa-miR-542-5p	42612	0.002	1.98 vs 17.36
hsa-miR-363	1E+21	1E−05	0.1 vs 70.04	hsa-miR-142-5p	5E+35	0.003	65.06 vs 183.66
hsa-miR-564	3E+12	1E−05	1.7 vs 42.93	hsa-miR-193a-5p	534	0.002	0.1 vs 9.16
hsa-miR-622	6E+08	2E−05	1.6 vs 30.84	hsa-miR-198	293	0.002	0.1 vs 8.30
hsa-miR-1224-5p	4E+26	2E−05	0.1 vs 88.40	hsa-miR-22	3E+37	0.007	323.77 vs 448.43
hsa-miR-223*	3E+15	4E−05	12.66 vs 96.29	hsa-miR-663	6E+15	0.002	0.1 vs 52.43
hsa-miR-199b-5p	10E+16	3E−05	2.7 vs 59.19	hsa-miR-30e*	4E+09	0.004	44.23 vs 76.03
hsa-miR-222	2E+15	3E−05	0.1 vs 50.99	hsa-miR-191	10	0.002	0.1 vs 3.37
hsa-miR-582-5p	2E+24	3E−05	0.1 vs 80.94	hsa-miR-588	68	0.002	0.1 vs 6.18
hsa-miR-145	7E+20	4E−05	26.26 vs 95.56	hsa-miR-134	8E+33	0.002	19.53 vs 132.10
hsa-miR-143	2584	4E−05	0.1 vs 11.43	hsa-miR-186	5E+10	0.002	26.64 vs 62.05
hsa-miR-557	1E+08	4E−05	0.1 vs 26.68	hsa-miR-760	4E+09	0.003	3.21 vs 35.11
hsa-miR-664*	10E+26	4E−05	0.1 vs 89.72	hsa-miR-135a*	106	0.003	0.1 vs 6.82
hsa-miR-31	18	5E−05	0.1 vs 4.26	hsa-miR-659	192232	0.003	0.1 vs 17.65
hsa-miR-98	5E+17	6E−05	0.1 vs 58.88	hsa-miR-361-3p	9E+09	0.004	11.98 vs 44.97
hsa-miR-199a-5p	3E+22	7E−05	0.1 vs 74.59	hsa-miR-518a-5p	19	0.005	0.1 vs 4.34
hsa-miR-1183	4E+21	9E−05	0.1 vs 71.99	hsa-miR-650	16	0.005	0.1 vs 4.11
hsa-miR-1300	10E+10	9E−05	0.1 vs 36.60	hsa-miR-197	5E+22	0.006	33.08 vs 108.50
hsa-miR-1225-3p	8565	0.0002	0.1 vs 13.16	hsa-miR-196b	16	0.007	0.1 vs 4.13
hsa-miR-149*	13087	0.0001	0.1 vs 13.77	hsa-miR-664	8E+06	0.007	2.61 vs 25.61
hsa-miR-155	2E+27	0.0001	0.1 vs 90.52	hsa-miR-595	4602	0.009	0.1 vs 12.26
hsa-miR-513a-5p	5E+37	0.0002	42.98 vs 168.19	hsa-miR-320c	1E+15	0.009	162.57 vs 212.83
hsa-miR-601	8E+09	0.0001	0.1 vs 32.98	hsa-miR-874	2E+07	0.009	0.1 vs 24.51
hsa-miR-181c	515352	0.0002	0.1 vs 19.07				

Shown is the fold change difference expression of K562 cells compared to healthy blood samples (second column from left), and p-value (third column from left), for each presented miRNA (first column from left). The last column displays raw experimental expression data presented as log2 values (0.1 is background level).

**Table 2:** Summary of miRNAs **upregulated** in K562 cells as compared to healthy blood samples.

miRNA ID	Fold change	p-value	Raw log2 values (mean K562 vs blood)
hsa-miR-495	1E+24	9E−05	85.83 vs 5.64
hsa-miR-493*	1E+31	4E−05	104.17 vs 1.00
hsa-miR-365	6E+12	0.0001	92.17 vs 49.86
hsa-miR-128	4E+15	0.0003	99.18 vs 47.43
hsa-miR-543	1E+08	0.0004	27.14 vs 0.1
hsa-miR-382	4E+09	0.0032	39.34 vs 7.29
hsa-miR-409-3p	2E+34	0.0004	130.16 vs 16.14
hsa-miR-154	2E+09	0.0007	30.63 vs 0.1
hsa-miR-654-3p	1E+34	0.0001	120.35 vs 7.06
hsa-miR-193b	6E+22	0.0003	75.81 vs 0.1
hsa-miR-409-5p	5E+08	4E−05	28.83 vs 0.1
hsa-miR-379	7E+25	0.002	85.99 vs 0.1
hsa-miR-432	2E+11	0.003	49.95 vs 12.23
hsa-miR-21*	1E+06	0.0007	43.76 vs 23.43
hsa-miR-18a*	791	0.0007	9.73 vs 0.1
hsa-miR-181b	1E+14	0.005	97.43 vs 50.69
hsa-miR-329	4E+10	0.003	53.21 vs 0.1
hsa-miR-105	6864	0.003	12.85 vs 0.1
hsa-miR-410	4E+20	0.003	76.29 vs 7.78
hsa-miR-96	9E+16	0.004	56.35 vs 0.1
hsa-miR-758	1E+16	0.003	53.49 vs 0.1
hsa-miR-411	5E+08	0.007	28.85 vs 0.1
hsa-miR-431	1E+18	0.004	66.07 vs 5.99
hsa-miR-299-3p	5E+14	0.003	48.98 vs 0.1
hsa-miR-337-5p	5E+13	0.004	45.45 vs 0.1
hsa-miR-92a-1*	7E+18	0.001	62.59 vs 0.1
hsa-miR-151-3p	8E+15	0.002	95.62 vs 42.78
hsa-miR-431*	1E+16	0.001	53.52 vs 0.1
hsa-miR-323-3p	8E+11	0.008	39.69 vs 0.1
hsa-miR-154*	3E+34	0.002	114.72 vs 0.1
hsa-miR-320a	7E+28	0.006	192.48 vs 96.57
hsa-miR-9	1E+15	0.007	50.01 vs 0.1
hsa-miR-338-5p	7132	0.009	18.90 vs 6.1
hsa-miR-335*	12549	0.009	13.71 vs 0.1
hsa-miR-486-3p	6E+06	0.002	22.58 vs 0.1

Shown is the fold change difference expression of K562 cells compared to healthy blood samples (second column from left), and p-value (third column from left), for each presented miRNA (first column from left). The last column displays raw experimental expression data presented as log 2 values (0.1 is background level).

**Figure 2:**
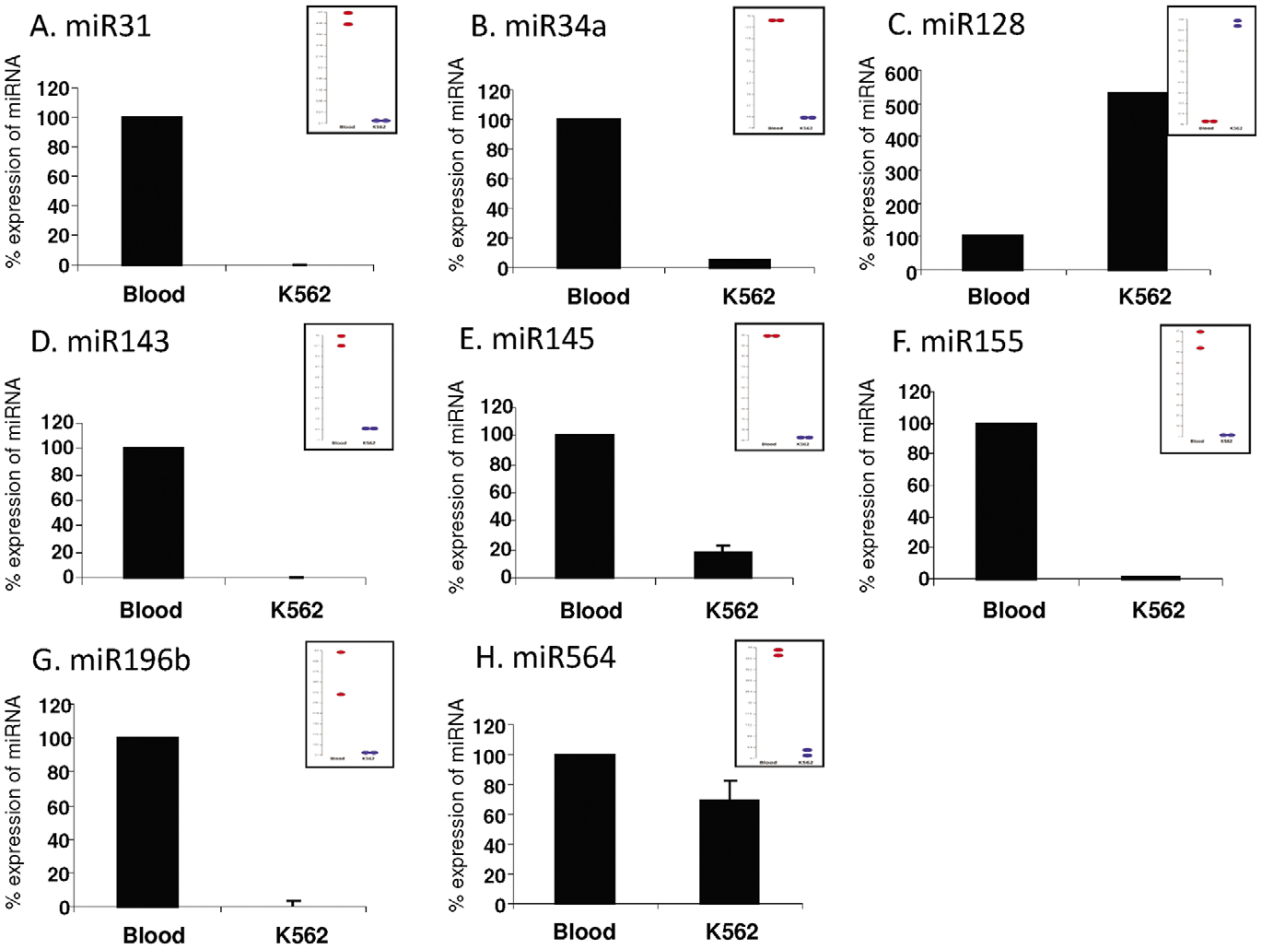
Real-time RT-PCR validation of microarray results, presenting changes in miRNA expression in K562 cells as compared to a pooled sample of healthy blood. The expression of A. miR-31, B. miR-34a, D. miR-143, E. miR-145, F. miR-155, G. miR-196b and H. miR-564 was downregulated whereas the expression of C. miR*-*128 was upregulated in K562 cells compared to healthy blood. The histograms show percentage of miRNA expression normalized to U6-snRNA±SEM from at least 3 experiments. Insets show microarray expression results for the specified miRNAs (Blood: red circles; K562: blue circles).

In order to validate the microarray data and to ensure that the variations observed were biological and not technical we carried out Taqman miR–qRT-PCR analyses of several miRNAs with altered expression between the K562 cell sample and the normal pooled blood sample. We chose a subset of miRNAs for validation; some of which are known to be altered in cancer while others are potential regulators of cancer/growth pathways in the cell. Each miRNA was quantified in the sample and its expression level was normalized to that of the U6-snRNA, which was used as reference. The miRNA expression patterns from the K562 cells and from the normal pooled blood sample were consistent between the microarray and Taqman measurements. Overall, with the aid of miRNA-based microarray, miR–qRT-PCR and preliminary bioinformatics analysis, we identified 8 miRNAs with statistically significant differential expression between K562 cells and healthy controls: 7 of them were downregulated in K562 cells (miR-31, miR-34a, miR-143, miR-145, miR-155, miR-196b and miR-564) and one was upregulated (miR-128) in K562 cells (Figure 2).

**Figure 3:**
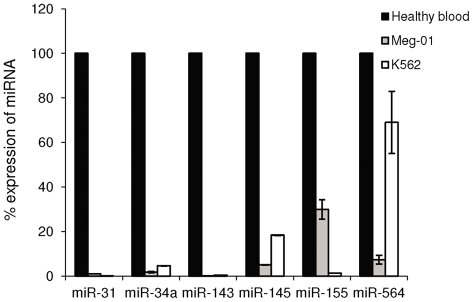
Expression analysis of miRNAs in CML-derived cell lines as compared to a pooled sample of healthy blood. Expression analysis of miR-31, miR-34a, miR-143, miR-145, miR-155 and miR-564 in Meg-01 and K562 cell lines and in healthy blood samples was analyzed by miR–qRT-PCR. Shown is the percentage of miRNA expression normalized to U6-snRNA±SEM from at least 3 experiments.

To further test whether these miRNAs could be implicated specifically in the pathogenesis of CML, we analyzed the expression of the deregulated miRNAs in an additional CML cell line; Meg-01 and in a different hematological, non-CML cell line; HL60. Of the 7 miRNAs found to be downregulated in K562, 6 (miR-31, miR-34a, miR-143, miR-145, miR-155, and miR-564) showed a low expression pattern in Meg-01 cells (Figure 3). The expression of 2 of these 6 miRNAs, miR-143 and miR-145, was low in HL60 cells as well indicating that these miRNA are probably not associated exclusively with CML (data not shown). The expression of the remaining 4 miRNAs (miR-31, miR-34a, miR-155, and miR-564) was high in healthy blood and in HL60 and low in K562 and in Meg-01, indicating a higher likelihood of these miRNAs to be involved in CML pathogenesis.

### BCR-ABL kinase activity induces downregulation of miR-31, miR-155, and miR-564 in CML

**Figure 4:**
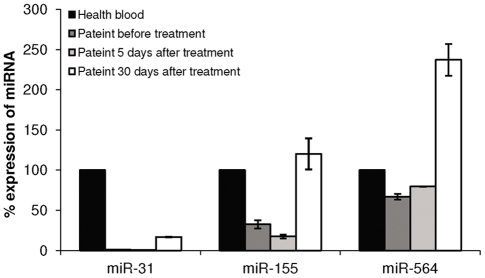
Expression analysis of miRNAs in a CML patient before and during treatment with imatinib. Expression analysis of miR-31, miR-155 and miR-564 in a CML patient at diagnosis an on day 5 of imatinib treatment at a dose of 100 mg/day (for the first week of treatment) and on day 30 of imatinib treatment at a dose of 400 mg/day (from the fourth week of treatment and thereafter). Expression was analyzed by miR–qRT-PCR. Shown is the percentage of miRNA expression normalized to U6-snRNA±SEM from at least 3 experiments.

To support the notion that the above mentioned miRNAs are implicated in CML, we tested the expression of these miRNAs in a CML patient. The analysis confirmed the statistically significant difference in the expression of 3 of the 4 miRNAs (miR-31, miR-155, and miR-564) between leukocytes from a CML patient and those from healthy donors (Figure 4). As CML is characterized by the presence of the BCR-ABL protein, we reasoned that the abnormal expression of these 3 miRNAs observed in the CML patient could be dependent on the kinase activity of BCR-ABL. To test this hypothesis, the expression of these miRNAs was analyzed in leukocytes of a CML patient before and during treatment with imatinib. Whereas these miRNAs showed virtually no change in their expression level on day 5 of imatinib treatment at a dose of 100 mg/day (for the first week of treatment), 30 days of treatment with imatinib at a dose of 400 mg/day (from the fourth week of treatment and thereafter) induced an upregulation of all 3 miRNAs (Figure 4).

### Target and pathway analysis of the identified miRNAs

MiRNAs can regulate numerous target genes and therefore have the potential to modulate multiple pathways. Several databases based on various algorithms are available for predicting the targets of selected miRNAs. In order to determine potential target genes and signaling pathways implicated in CML, we analyzed predicted targets of the 3 miRNAs differentially expressed in this study using the TargetScan Human 5.1 database (Table 3).

**Table 3:** Putative target genes of the deregulated miRNAs as predicted by Target Scan analysis.

	miR-31 ↓	miR-155 ↓	miR-564 ↓
**CBL**	CBL	CBL	
**E2F2**	E2F2	E2F2	
**E2F3**			E2F3
**Cyclin D1**		Cyclin D1	
**TGFβR2**		TGFβR2	
**K-ras**		K-ras	
**PIK3R1**		PIK3R1	
**SOS1**		SOS1	
**Akt2**			Akt2
**p-value**	**0.58**	**9.1E−07**	**3.04E−05**

Arrows indicate the expression of the miRNA in CML cell lines and patients in reference to its expression in non-CML cell lines and healthy blood. Abbreviations are as follows: CBL, Casitas B-lineage lymphoma; TGFβR, transforming growth factor-beta receptor; K-ras, Kirsten rat sarcoma; PIK3R1, phosphatidylinositol 3-kinase regulatory subunit alpha; SOS1, son of sevenless homolog 1.

At this point, we sought to correlate the expression of some of the significantly deregulated miRNAs in K562 with some of their putative targets as predicted by our bioinformatics analysis. For this purpose, we performed real-time PCR analysis on CBL, E2F3, cyclin D1, K-ras and Akt2; five of the most promising target proteins to be involved in CML.

**Figure 5:**
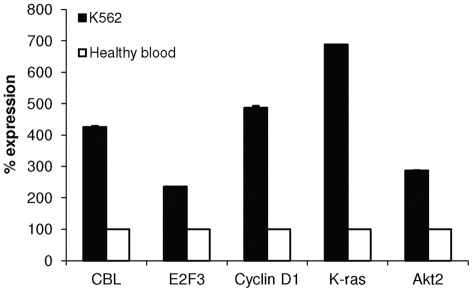
Expression of putative targets of the derugulated miRNAs. Expression of CBL, E2F3, cyclin D1, K-ras and Akt2 was analyzed by real-time PCR in K562 cells and in healthy blood samples**.** Shown is the percentage of miRNA expression normalized to GUSB RNA±SEM from at least 3 experiments.

CBL which is involved in cancer initiation and progression and is believed to form a multi-protein complex with BCR-ABL [26], [27] is a putative targets of miRs-31 and miR-155. Cyclin D1 and K-ras which have been shown to cooperate with ABL oncogenes to induce full in-vitro and in-vivo transformation [28], [29] are putative targets of miR-155. E2F3 and Akt2 expression is important for BCR-ABL clonogenic activity and are putative targets of miR-564 [30], [31]. Given the downregulated expression of these miRNAs in K562 cells, we hypothesized that the levels of the above mentioned targets would be elevated in this cell line. Indeed, we found that the expression level of CBL, E2F3, cyclin Dl, K-ras and Akt2 to be significantly higher in K562 cells compared to normal blood samples (Figure 5).

**Table 4:** Ingenuity analysis of miRNA predictive pathways.

	miR-31/miR-155/miR-564
**Disease**	Colorectal cancer
	CML
**Molecular pathway**	T/B cell receptor signaling pathway
	MAPK signaling pathway
	ErbB signaling pathway
	mTOR signaling pathway
	VEGF signaling pathway

**Figure 6:**
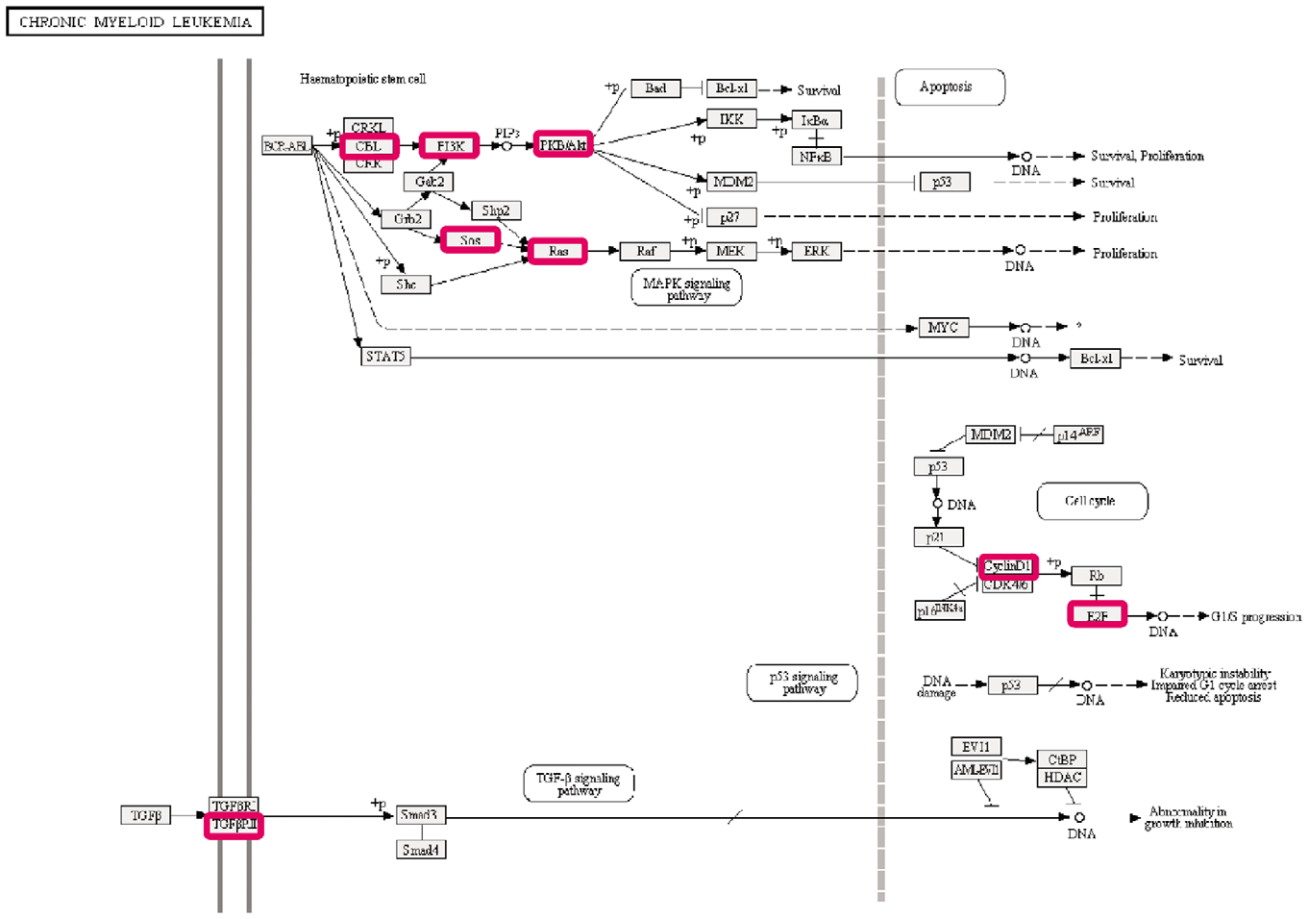
KEGG pathways of deregulated miRNAs predicted targets in CML. This figure presents cell survival and growth signaling pathways within a CML cell. Red squares represent proteins encoded by genes targeted by the deregulated miRNAs. We have shown that the miRNAs regulating these target genes are downregulated in CML. As a result, these target genes are probably upregulated leading to increased cell survival and induced of uncontrolled cell proliferation.

**Figure 7:**
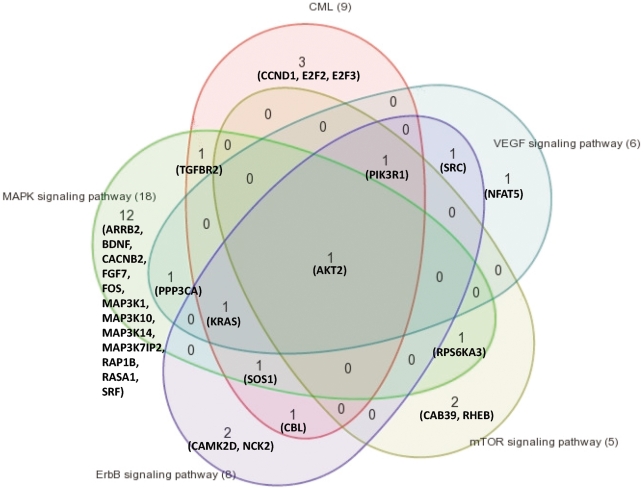
Overlap of predicted target genes. A Venn diagram showing the overlap between predicted targets of CML-related miRNAs and miRNAs related to the different pathways that are predicted to be deregulated in our study.

Based on these results, we explored biological significance, molecular functions and possible diseases affected by the predicted targets of the 3 deregulated miRNAs, by using Ingenuity Systems pathway analysis software (Table 4). Reassuringly, the analysis identified cancer as the main disease associated with expression of these 3 deregulated miRNAs, and specifically CML as one of the major neoplasms linked to these miRNAs. Colorectal cancer was an additional prominent disease found to be related to these deregulated miRNAs. The MAPK, ErbB, mTOR and VEGF signaling pathways were the main molecular pathways related with these expression patterns. In addition to Ingenuity Systems pathway analysis, KEGG pathway analysis also demonstrated CML as one of the main diseases associated with these miRNAs. Figure 6 shows the molecular pathways, as analyzed by the KEGG pathway database, which may be deregulated in CML due to improper expression of the target genes summarized in Table 3, as a result of the deregulated miRNAs. These pathways correlate with cell survival and uncontrolled cell proliferation. Next, we wanted to hypothetically establish possible logical relations between the top scoring pathways identified by our analyses. Interestingly, according to Venn diagram analysis, there is an overlap between the CML-related miRNAs and the MAPK, ErbB, mTOR and VEGF signaling pathways-related miRNAs (Figure 7).

Overall, the data derived from our miRNA profiling and bioinformatics analysis support the notion that the miRNAs identified in this study may have a pivotal role in CML. Nevertheless, while these data point to a central disease, the precise molecular pathways targeted by these miRNAs are variable implying a high level of complexity of miRNA target selection and regulation.

## Discussion

Distinct miRNA expression signatures are known to participate in regulatory pathways involved in the development and progression of many types of tumors. Accordingly, to date, all tumors tested have shown significantly different miRNA profiles compared with normal cells from the same tissue. These miRNA expression signatures have been reported to be associated with diagnosis, prognosis and response to treatment [32]. Since miRNAs affect a wide range of molecular pathways, studying miRNA expression in human cancer may provide a better understanding of the molecular pathways contributing to cancer pathogenesis and progression.

We utilized miRNA microarray analysis and miRNA real-time PCR to detect miRNAs whose expression is deregulated in CML. To demonstrate whether different sets of miRNAs may properly classify our groups of interest, we performed unsupervised cluster analysis using Partek Genomics Suite software. The unsupervised cluster analysis generated a tree that clearly separated healthy blood samples from K562 cells suggesting that this miRNA profile may serve as a marker differentiating CML patients from healthy individuals. In general, a higher percentage of miRNAs were downregulated rather than upregulated in K562 cells as compared to normal blood samples. This is in agreement with the acknowledged concept that overall downregulation of miRNA expression is characteristic of many cancers, as compared to their normal tissue counterparts [13], [14].

In this study we identified 3 miRNAs (miR-31, miR-155, and miR-564) to be abnormally downregulated in CML cell lines and in patients with CML as compared to non-CML cell lines and to healthy blood samples thus providing support to our hypothesis that miRNAs could be implicated in CML. Moreover, our data suggest that the low expression of these miRNAs is dependent on BCR-ABL tyrosine kinase activity as demonstrated by their increased expression in leukocytes of an imatinib treated patient. Further studies are required to address the molecular mechanism by which BCR-ABL is responsible for the downregulated expression of these miRNAs and whether this downregulation is essential for the transforming role of BCR-ABL in CML.

One miRNA that we found to be downregulated in CML is miR-155. Several lines of evidence suggest that this miRNA may have an important role in normal hematopoiesis [33]. Similar to our quest for defining a signature expression pattern associated with CML, Calin et al. defined a unique miRNA signature linked with prognostic factors and disease progression in CLL. This unique signature was composed of 13 miRNA, among them was miR-155 [34]. However, in contrast to our findings, this miRNA has been reported to accumulate in several lymphoma subtypes, especially in diffuse large B cell lymphoma [35], Hodgkin lymphoma [36] Burkitt lymphoma [37] and in CLL [38]. These discrepancies may be due to differences in the malignancy investigated. They could also be derived from technical differences influenced by the type of technique used for the identification of the relevant miRNA. Also, differences in the normal control used could also have influenced the outcome of the miRNA profiling analysis.

MiR-31, which was also shown to be downregulated in our cell model, is one miRNA out of a cluster of miRNAs that have been assigned to 9 p (miRBase, http://microrna.sanger.ac.uk/) and documented as recurrently deleted when 9 p instability is present, such as witnessed in many CML cases [39]. MiR-31 has recently been shown to concurrently repress the expression of multiple prometastatic target genes thereby inhibiting several distinct aspects of the invasion-metastasis cascade including motility, invasion and resistance to anoikis. As evidence, overexpression of miR-31 in otherwise-aggressive breast tumor cells suppresses metastasis. Whereas inhibiting miR-31 in-vivo allows otherwise-nonaggressive breast cancer cells to metastasize [40]. In contrast to these and to our results, miR-31 was shown to be upregulated in colorectal tumor cells in comparison to non-tumor adjacent mucosa and in plasma of oral squamous cell carcinoma [41], [42]

Expression of miR-564, which we found to be downregulated in our setting, has not been evaluated previously in CML, and the published data regarding this miRNA seems to be inconsistent. It was shown to be downregulated in multiple myeloma, upregulated in CLL and baring no significant change in expression level in hairy cell leukemia compared with normal levels [43].

Ingenuity Systems pathway analysis and KEGG pathway analysis identified CML as one of the major neoplasms linked to these deregulated miRNAs. This analysis also recognized the MAPK, ErbB, mTOR and VEGF signaling pathways as the main molecular pathways related with these expression patterns. These signaling pathways usually participate in a wide range of cellular processes such as growth, proliferation, differentiation and apoptosis. These pathways have continuously been reported to play an important role in hematological malignancies in general and in CML in particular. For instance, BCR-ABL has been shown to activate mTOR and MAPK signaling cascades. These pathways are important components of the aberrant signaling induced by BCR-ABL and contribute to BCR-ABL leukemogenesis. (reviewed in [44], [45], [46], [47]).

In order to determine potential genes implicated in CML, we analyzed predicted targets of the 3 miRNAs differentially expressed using several different algorithms. Nine predicted targets of the 3 deregulated miRNAs have a p-value≤0.005. Most of these targets have been published as related to CML [30], [48], [49], [50], [51]. We observed a downregulation in the expression of the miRNAs targeting Akt2, CBL, E2F2, E2F3, Cyclin Dl, K-ras, PIK3R1, SOS1 and TGFβR2 in CML cell lines and patients as compared to healthy blood. We can therefore hypothesize that the levels of these targets would be elevated in CML cell lines and patients. To begin to confirm the biological effects of these deregulated miRNAs, the expression of CBL, E2F3, cyclin Dl, K-ras and Akt2 was studied. Interestingly, the expression of these proteins was indeed found to be upregulated in K562 cells compared to healthy cells.

Among the possible targets for these miRNAs we found several to mediate transformation of primary human hematopoietic cells. We found that miRs-31 and -155, which were downregulated in our setting, potentially target CBL, which is involved in cancer initiation and progression and was originally discovered as an oncogene which induces B-cell and myeloid leukemias in mice [52]. The expression of this target protein was found to be elevated in K562 cells as compared to control cells. The emerging view is that BCR-ABL assembles a multi-protein complex whose signaling output leads to cellular transformation. Among other proteins, CBL has been identified as participating in this multi-protein complex and as being phosphorylated by BCR-ABL. As a result of these interactions many intracellular signaling pathways, participating in cellular proliferation and survival, are activated, including the K-ras, Akt2 and STAT pathways [26], [27].

E2F3 is an addition protein known to be important for BCR-ABL leukemogenic potential [30]. E2F3 mRNA level has been reported to be higher in BCR-ABL-expressing cell lines compared with non BCR-ABL-expressing cells, and decreases upon inhibition of BCR-ABL kinase activity. Eiring et al have suggested that E2F3 plays a major role in controlling BCR-ABL oncogenic activity and that its expression is required for in-vivo BCR-ABL leukemogenesis. In addition, they show enhanced proliferation to be a characteristic of CML-blast crisis cells, where E2F3 mRNA levels are high but not of CML-chronic phase cells, where E2F3 mRNA levels are low [30]. Our data reveal that miR-564, a putative regulator of E2F3, is downregulated in CML. It is possible that the reduced expression of miR-564 leads to an enhanced expression of E2F3 which assists in the oncogenic activity of BCR-ABL and the growth advantage seen in CML-blast crisis cells.

An additional protein shown to cooperate with BCR-ABL to induce full cellular transformation is cyclin D1. Afar et al have shown that cells overexpressing cyclin Dl (and not cyclin E) and BCR-ABL formed more numerous and larger colonies in soft agar than cells expressing ABL alone [28]. The upregulated expression of cyclin D1 witnessed in K562 cells has been proposed to be achieved through its highly active promoter in these cells [53]. Alternatively, the enhanced expression of cyclin D1 in these cells could be attributed to the reduced expression of miR-155 putatively regulating it.

Ras signaling pathways are activated by a spectrum of hematopoietic cytokine receptors and therefore play important roles in the proliferation and enhanced survival of hematopoietic progenitors. For instance, genetic and biochemical data argue that Ras activation plays a central role in leukemogenic transformation by BCR-ABL [29]. Nevertheless, activating K-ras mutations are detected more often in solid tumors and are a rare event in hematological malignancies in general and in CML in particular [54], [55]. In the absence of oncogenic Ras mutations, alternative mechanisms activating Ras, such as oncogenic activation of upstream tyrosine kinases as seen in AML cells [56], [57], [58], [59] and loss-of-function mutations in the Ras-GAP protein; neurofibromatosis 1 as seen in juvenile myelomonocytic leukemia (JMML) [60], have been suggested. We found the expression of miR-155, a putative negative regulator of K-ras, to be impressively downregulated in CML cell lines and patients. A decrease in miR-155 may serve as an additional mechanism leading to deregulated expression of oncogenic Ras.

Akt2 and Akt3, are genomically amplified in several ovarian, pancreatic and breast cancers [61], [62]. In addition, in a study performed to elucidate the molecular mechanisms underlying CML disease progression, whole-genome profiling was performed to identify recurrent submicroscopic gains and losses of genetic material. In this study, gains for Akt2 were observed [31]. These data propose that overexpression of Akt2 may contribute to the CML malignant phenotype or to malignant disease progression. We also detected a high expression level of Akt2 in K562 cells. We however suggest that this upregulated expression might be due to decreased expression of miR-564, putatively regulating Akt2, in K562 cells.

In this study we identified 3 miRNAs deregulated in CML cell lines and patients versus non-CML cell lines and healthy cells. The presented data suggest that numerous miRNA genes are implicated in the regulation of multiple pathways in hematopoiesis and when aberrantly expressed may promote CML predisposition, initiation and progression. Our identification of CML-related miRNAs and their putative targets can be used as a basis to explore the functions of these miRNAs. Nevertheless, further large scale studies and comprehensive analyses are imperative to validate the role of these miRNAs in CML and to determine whether selective miRNA targeting might modify disease phenotype and outcome. In addition, studies including introduction of exogenous miRNAs or antisense to miRNAs are needed to confirm the direct relationship between each miRNA and its putative target. Ultimately, such studies will have a guiding significance in research and clinical applications and will hopefully aid in the development of novel therapeutic strategies and in determining if these deregulated miRNAs and their targets could be used as diagnostic or prognostic indicators.
